# OP20 Technical Validation Of An Environmental Model: A Multidisciplinary Checklist To Guide Environmental Criteria In Health Technology Assessment

**DOI:** 10.1017/S0266462325100780

**Published:** 2025-12-29

**Authors:** Melissa Pegg

## Abstract

**Introduction:**

The climate crisis is a health crisis. The health technology assessment (HTA) community is lacking appropriate support and resources to adequately support the evaluation of health technology environmental sustainability. There is a need for reporting guidance to help authors, journal editors, and peer reviewers in their identification and interpretation of sustainability outcomes within HTA. The goal of the Sustainability in HTA (SUSHTA) checklist is to promote key collaboration that aids sustainable healthcare development.

**Methods:**

The key aims of the SUSHTA checklist were to validate an environmental model created by industry and provide recommendations to support appropriate framing and reporting of the environmental data. International methodological guideline sources, HTA principles including reproducibility and transparency, and best practice health economics model validity frameworks were used with an interdisciplinary, multidimensional approach. The framework supports pertinent, high quality evidence that extends beyond conventional sources. The checklist has been used as a tool to facilitate collaboration with industry in reporting an information conduit to further aid data sharing and reduce data paucity among HTA agencies and to support healthcare decision-making.

**Results:**

The SUSHTA checklist is holistic and comprehensive through its interpretation of environmental models, including validity checks of terminology, labeling and referencing, model justification, data collection and calculation techniques, assumptions, sensitivity and scenario analysis, and uncertainty modeling. The framework also supports applying the same principles to environmental impacts other than greenhouse gas emissions. Therefore, this framework can be applied to assess a wider range of environmental outcomes (data) for human health impact (disability-adjusted life years), resource use (e.g., water or fossil fuel use), and biodiversity loss (number of species lost per year). The checklist is generalizable and integrates well with healthcare system frameworks.

**Conclusions:**

The SUSHTA checklist is primarily intended for researchers to recommend the minimum amount of information required for reporting environmental data. However, familiarity with reporting requirements will be useful for analysts when planning studies. It may also be useful for HTA agencies seeking guidance on reporting, as there is an increasing emphasis on transparency and reproducibility in more sustainable decision-making processes.

